# Hydroalcoholic Extract and Ethyl Acetate Fraction of* Bixa orellana* Leaves Decrease the Inflammatory Response to* Mycobacterium abscessus* Subsp.* massiliense*

**DOI:** 10.1155/2018/6091934

**Published:** 2018-10-02

**Authors:** José Lima Viana, Adrielle Zagmignan, Luís Felipe Lima Lobato, Afonso Gomes Abreu, Luís Cláudio Nascimento da Silva, Joicy Cortez de Sá, Cristina de Andrade Monteiro, João Henrique Ghilardi Lago, Letícia Machado Gonçalves, Rafael Cardoso Carvalho, Lídio Gonçalves Lima Neto, Eduardo Martins de Sousa

**Affiliations:** ^1^Pós-Graduação em Biologia Parasitária, Laboratório de Imunologia e Microbiologia das Infecções Respiratórias, Universidade Ceuma, São Luís, Brazil; ^2^Pós-Graduação em Biologia Parasitária, Laboratório de Micologia Médica, Programa de Mestrado em Biologia Parasitária, Universidade Ceuma, São Luís, Brazil; ^3^Centro de Ciências Naturais e Humanas, Universidade Federal do ABC, São Paulo, Brazil; ^4^Pós-graduação em Odontologia, Laboratório de Odontologia, Universidade Ceuma, São Luís, Brazil; ^5^Programa de Pós-Graduação em Ciências da Saúde, Universidade Federal do Maranhão, São Luís, Brazil

## Abstract

The incidence of infections caused by rapidly growing mycobacteria (RGM), especially* Mycobacterium abscessus *subsp.* massiliense *(*Mabs*), is increasing worldwide. Severe infections are associated with abscess formation and strong inflammatory response. This study evaluated the antimicrobial and anti-inflammatory activities of a hydroalcoholic extract (BoHE) and ethyl acetate fraction (BoEA) of* Bixa orellana *leaves. Antimicrobial activity was evaluated by broth microdilution to determine the minimum inhibitory (MIC) and the minimum bactericidal (MBC) concentrations. Cytotoxicity was evaluated using erythrocytes and RAW 264.7 cells. Nitric oxide (NO) was assayed in stimulated RAW 264.7 cells, and inflammatory cell migration and acute toxicity were evaluated in a* Mabs*-induced peritonitis mouse model. The compounds present in BoEA were identified by high performance liquid chromatography and mass spectrometry (HPLC-MS). The MIC and MBC values were 2.34 mg/mL and 37.5 mg/mL for BoHE and 0.39 mg/mL and 6.25 mg/mL for BoEA. The extracts did not induce significant toxicity in erythrocytes and RAW 264.7 cells. High levels of NO induced by* Mabs* were decreased by treatment with both extracts. The anti-inflammatory activity was confirmed* in vivo *by significant reduction of the cell migration to the peritoneum following BoHE and BoEA pretreatment. Animals treated with BoHE or BoEA did not show signs of acute toxicity in stomach, liver, and kidney. The chemical characterization of BoEA (the most active extract) revealed that kaempferol-3-O-coumaroyl glucose is its major component. The extract of* B. orellana *may be effective for treating infections caused by* Mabs.*

## 1. Introduction

The* Mycobacterium abscessus* complex is formed by* M. abscessus *subsp.* abscessus*,* M. abscessus *subsp.* massiliense*, and* M. abscessus *subsp.* bolletii* [1, 2]. These rapidly growing mycobacteria (RGM) cause hospital outbreaks of lung infections in patients with cystic fibrosis, chronic lung disease (bronchiectasis, nodules, and cavitations), postsurgical infections [3, 4], skin and soft tissue infections in immunocompromised patients [5, 6], and peritonitis in peritoneal dialysis patients [7].

An impermeable cell wall composed of peptidoglycans and mycolic acids makes* M. abscessus *subsp.* massiliense *and other RGM species naturally resistant to antimicrobials and disinfectants [8, 9] and creates challenges in the search for new treatments [10]. Acquired macrolide resistance may develop during treatment of* M. abscessus *subsp.* massiliense *lung infections and is conferred by mutations in the drug binding pocket of the 23S rRNA gene (*rrl*) at nucleotide positions 2058 and 2059 [11–13].

Previous studies have described* Bixa orellana* (also known as* urucum*) as a source of antimicrobial agents. The seeds of this plant are currently used in folk medicine to treat heart disease, gastrointestinal problems, respiratory infections, burns [14], diabetes, skin infections, fever, measles, gonorrhea, diarrhea, and asthma [15]. The antimicrobial action [16] and antioxidant activity [17] of the methanolic extract of* B. orellana* seeds and leaves against Gram-positive bacteria (e.g.,* S. mutans *and* S. sanguinis*) have previously been described. In addition, the antimalarial activity of essential oils and root extracts [18] and the anti-inflammatory effects of aqueous extracts of the leaves [19, 20] have been also reported.

This study evaluated the antimicrobial and anti-inflammatory activities of BoHE and BoEA leaf extracts of* B. orellana*, which is a plant widely used in Brazilian folk medicine to treat* Mycobacterium* infections and other inflammatory disorders [14]. The antimicrobial activity was tested against* M. abscessus *subsp.* massiliense *(*Mabs*), an emerging human pathogen implicated in hospital outbreaks with natural resistance to several last generation antimicrobials. The cytotoxic effects of BoHE and BoEA were assayed using human erythrocytes and murine RAW 264.7 cells. The acute toxicity and the* in vivo* anti-inflammatory activities of the extracts were evaluated in a murine peritonitis model induced by inactivated* Mabs*.

## 2. Materials and Methods

### 2.1. Collection of* B. orellana* Leaves and Preparation of Extract and Fractions

Fresh* B. orellana* leaves were collected (June–July 2016) and identified at the Ático Seabra Herbarium of Federal University of Maranhão (São Luís, Brazil; voucher specimen 1147). The cleaned leaves were oven dried at 40°C for 3 days, ground in a mill, and extracted for 24 h in 70% ethanol by maceration with agitation. The sample was filtered, and the resulting filtrate was concentrated in a rotary evaporator under low pressure at 50°C. The concentrate was lyophilized (named BoHE) and stored at -20°C until use. BoHE was submitted to liquid-liquid fractionation using hexane, chloroform, and ethyl acetate with a series of increasing polarity to produce hexane (BoHex), chloroform (BoCl), and ethyl acetate (BoEA) fractions. BoHE and BoEA were resuspended in a saline solution (0.9% NaCl).

### 2.2. Determination of Total Phenol and Total Flavonoid Contents

The content of total phenols was estimated using the Folin-Ciocalteau method for total phenols (Singleton et al., 1965), with some modifications. Samples of 20 *μ*L of each extract (1 mg/mL) and 100 *μ*L of the Folin-Ciocalteu reagent were mixed; and after 3 minutes of incubation at room temperature, 80 *μ*L of a solution of sodium bicarbonate (0.7 M) was added. The reaction was kept in the dark for 2 h at room temperature and the absorbance was measured at 735 nm using a microplate reader (BioTek UQuant MQX200). PBS (vehicle) was used as negative controls. Gallic acid was used as standard, and the results were calculated based on the calibration curve of gallic acid (10-100 mg/L) and expressed as mg equivalent of gallic acid per gram of extract (GAE /g extract).

The flavonoid content was determined according to the colorimetric method of aluminum chloride (Woisky and Salatino, 1998). The extracts were tested at the concentration of 1 mg/mL, and the quercetin was used as standard compound (10-100 mg/L). The sample (100 *μ*L) was mixed with 100 *μ*L of the reagent [2% aluminum chloride (AlCl_3_) in methanol]. After 1 h on incubation in the dark at room temperature, the absorbance was read at 420 nm. PBS was used as negative control. The results were expressed as mg equivalent of quercetin per gram of extract (mg QE/g).

### 2.3. RGM Strain

An isolate of* M. abscessus massiliense* (Go01) obtained from patients with hospital outbreaks of postoperative infections [21] was used in the study. The sample was generously provided by the Laboratory of Immunopathology of Respiratory Infections from the Institute of Tropical Pathology and Public Health, the Federal University of Goiás. The use of the clinical isolate was authorized by freely given written informed consent using a form approved by the institutional ethics committee (N°: 21357413.4.0000.5084).

### 2.4. Antimicrobial Susceptibility

Minimum inhibitory (MIC) and minimal bactericidal (MBC) concentrations were determined as described in the ATS/IDSA guidelines [5]. In brief, the extracts were diluted (from 200 mg/mL to 0.04 mg/mL) in microplates and microbial suspensions (at 2x10^3^ CFU/mL) were added. After 72 h of incubation at 37°C, a resazurin suspension (0.01% w/v) was added followed by a further incubation (16 h incubation at 37°C). A pink color indicated the presence of living bacteria and a purple color indicated dead bacteria. Amikacin was used as a control. The extract concentrations without visible growth were inoculated on MH agar plates. The MBC was determined after 5–7 days incubation at 37°C. The MBC was the lowest extract concentration that prevented 99.9% bacterial growth expressed in CFU/mL [22, 23].

### 2.5. Cytotoxicity* Assays*

#### 2.5.1. Hemolytic Activity

Heparinized blood was obtained from three healthy volunteers (types A, B or O serotypes) that were nonsmokers and had not taken any medication for at least 15 days. They were 18–35 years of age and had given written informed consent. The protocol (N° 1.570.437) was approved by the Research Ethics Committee of Ceuma University. Erythrocytes were obtained by centrifugation at 1500 rpm for 10 min immediately after collection, removal of the plasma, and washing three times in pH 7.4 phosphate buffered saline. After washing, a 1% erythrocytes suspension was prepared in PBS. Samples (0.4 mL) of BoHE (0.58 mg/mL to 9.36 mg/mL) or BoEA (0.09 mg/mL to 1.56 mg/mL) were added to 1.1 mL erythrocyte suspension. PBS and 0.05% Triton X-100 solution were used as negative and positive controls, respectively. After 60 min incubation at room temperature, the cell suspensions were centrifuged, and the concentration of hemoglobin in the supernatant was measured at 540 nm [24]. The result was reported as the mean of three independent assays. Hemolytic activity was expressed in relation to Triton X-100 and calculated using the following formula:

% hemolysis = [(As − Ab) × 100] / (Ac − Ab), where Ab is absorbance of control (blank without extract), As is absorbance in the presence of the extract, and Ac is absorbance in the presence of Triton X-100.

#### 2.5.2. Cytotoxicity towards Murine RAW 264.7 Cells

Murine RAW 264.7 cells were maintained in Dulbecco's high glucose modified Eagle's medium (DMEM) supplemented with 10% fetal bovine serum (Sigma-Aldrich), streptomycin 100 *µ*g/mL, and penicillin 100 UI/mL. After growth was established, cells were plated in 96-well plates at 2 x 10^5^ cells/well and treated with BoHE (1.17 mg/mL to 9.36 mg/mL) or BoEA (0.19 mg/mL to 1.56 mg/mL). DMEM and 0.05% Triton X were positive and negative controls, respectively. The plated were incubated at 37°C and 5% CO_2_. After 24 h, the medium of each well was removed and DMEN medium containing 5 mg/mL MTT (3-(4,5-dimethylthiazol-2-yl)-2,5-diphenyltetrazolium bromide; Sigma-Aldrich) was added to each well. The plates were incubated for 4 h at 37°C and 5% CO_2_ in the dark. The supernatants were discarded, and the formazan crystals were dissolved in 100 *µ*L dimethyl sulfoxide (DMSO). Absorbance was read at 540 nm using a microplate reader (Thermo Plate), and the results were expressed as a percentage of the maximal value of the positive control and reported as means of three independent assays ± the standard deviation [25, 26].

### 2.6. *In Vitro *Anti-inflammatory Assay

The effects of BoHE and BoEA on nitric oxide (NO) production by murine RAW 264.7 cells induced by* Mabs* were evaluated using Griess-based assay. For this, RAW 264.7 cells were plated at a density of 2 x 10^5^ cells/well in 96-well plates and infected with* Mabs* at 1 x 10^8^ CFU/mL. After 1.5 h, the cells were exposed to BoHE (1.17 mg/mL and 9.36 mg/mL) or BoEA (0.19 mg/mL to 1.56 mg/mL).* Mabs*-infected cells without any extract treatment were considered as positive control. Cells treated and incubated with 1 *μ*g/mL LPS (*E. coli* 0111 lipopolysaccharide; Sigma-Aldrich, Saint Louis, MO, USA) and 100 *μ*g/mL IFN-*γ* (Interferon-gamma; BD Pharmingen) were also used as positive control. After 24 h incubation at 37°C in 5% CO_2_, the supernatants were collected (50 *μ*L) and incubated with 50 *μ*L Griess reagent (1% sulfanilamide, 0.1% naphthyl ethylenediamine, and 2.5% phosphoric acid) for 10 min at room temperature in the dark, and the absorbance at 540 nm was compared with that of a standard curve derived from 0−300 *µ*M NO [26]. Results were expressed as *µ*M.

### 2.7. Animal Experimentation

#### 2.7.1. Animals

Female C57BL/6 mice 6 to 8 weeks of age, weighing from 20 g to 25 g, were housed in plastic cages at room temperature (23 ± 1°C) and a 12 h light-dark cycle and given balanced laboratory food and water* ad libitum*. All experimental procedures were conducted following the laboratory animal care standards of the Ceuma University Animal Experimentation and Use Committee (approval N° 107/14) in accordance with the UK Animals (Directive 2010/63/EU).

#### 2.7.2. Clinical Isolates and Preparation of Inocula

*Mabs* bacterial suspensions were adjusted to 1 × 10^8^ CFU/mL in pH 7.4 phosphate buffered saline with vigorous stirring to disperse cell clumps. The bacterial cells were heat killed at 90°C for 1 h.

#### 2.7.3. Experimental Design and Induction of Peritonitis by* Mabs*

Animals were randomly allocated into seven groups (*n= *5-6 animals) that received a single oral dose of 0.9% saline solution at 1 mL/kg (groups I and II); BoHE at 50 mg/kg (group III) or 150 mg/kg (group IV); BoEA at 50 mg/kg (group V) or 150 mg/kg (group VI); and 5 mg/kg dexamethasone (group VII). After 1 h of treatment, the animals from groups II to VII received intraperitoneal injections of 0.1 mL 1×10^7^ CFU heat killed* Mabs*.

#### 2.7.4. Determination of Cell Migration to Peritoneal Cavity

Peritoneal liquid was obtained 4 h and 24 h after induction of peritonitis. For this, each animal received 80 mg/kg ketamine hydrochloride and 10 mg/kg xylazine hydrochloride. Lavage was performed with the introduction of 3 mL of 1 mM EDTA in PBS into the abdominal cavity with a sterile, disposable syringe, and needle. The aspirated contents were transferred to a tube. The leukocytes present in the peritoneal liquid were counted in a Neubauer chamber after a 1:2 v/v dilution in Turk's solution. The differential leukocyte count was performed in a 100 *µ*m hanging drop of sample obtained by cytocentrifugation at 600 rpm for 10 min. The slides were Giemsa stained, and the 100 cells were counted by optical microscopy at 1000 × using an oil immersion objective.

#### 2.7.5. Acute Toxicity Assay

Stomach, liver, and kidneys were in fixed 10% paraformaldehyde, embedded in paraffin, sectioned at 5 *μ*m, and stained with hematoxylin and eosin (H&E). The slides were evaluated by optical microscopy (Axio Imager Z2; Carl Zeiss, Oberkochen, Germany), at 40× to 400× increments, and ten fields per slide were evaluated by an experienced pathologist for the presence or absence, distribution, and severity of histological changes.

### 2.8. HPLC-DAD-ESI-IT/MS Analysis

The chemical constituents of BoEA were analyzed in a high-performance liquid chromatography (HPLC) using a LC-10AD system (Shimadzu, Japan) equipped with a photodiode array detector (DAD) and coupled to an Esquire 3000 Plus ion-trap mass spectrometer (Bruker Daltonics, Bremen, Germany), using electrospray ionization (ESI) using argon as collision gas and 80 eV as collision energy. Separation was performed using Phenomenex Kinetex C-18 column (250 × 4.6 mm, 5 *µ*m; Torrance, CA, USA). The column oven was maintained at room temperature. HPLC was set up with an elution gradient as follows: 0−2 min, 5% B; 2−10 min, 5−25% B; 10−20 min, 25−40% B, 20-30 min, 40-50% B, 30-40 min, 50-60% B, 40-50 min. Acetic acid (2%) in Milli-Q water was used as mobile phase A and methanol was used as mobile phase B.

The injection volume consisted of 25 *µ*L of reconstituted sample at 5 mg/mL and a flow rate of 0.6 mL/min. Detection was by a diode array detector (DAD) at 200–500 nm and a direct mass spectrometry/mass spectrometry method in negative electrospray (−ESI) mode with the detector voltage maintained at 4.0 kV, an ion source of 40 V, and capillary temperature of 320°C. The nebulizing gas was nitrogen (N_2_) flowing at 7 mL/min and sheath gas provided at a pressure of 27 psi, and helium was used as the four collision gas. The analyses were performed using full-scan mass spectra and data-dependent MS2 scans from 100 to 2000 m/z. The compounds were identified on the basis of their molecular ion mass fragmentation. The mass spectra were compared with those previously reported [27].

### 2.9. Statistical Analysis

Data were presented as means ± standard variation (SD) or percentages. The normality of distributions was determined by the Shapiro-Wilk test, and the differences between groups were evaluated by analysis of variance (ANOVA) followed by Tukey's multiple comparison test using the Graph Prism 6.0 software. P-values < 0.05 were considered significant.

## 3. Results

### 3.1. *B. orellana* Extracts Have Antimicrobial Activity against* M. abscessus massiliense*

*M. abscessus massiliense* (Go01) was susceptible to the* B. orellana* leaf extracts. The BoHE showed a MIC of 2.34 mg/mL and the MBC was 37.5 mg/mL, while MIC found in BoEA was 0.39 mg/mL and the MBC was 6.25 mg/mL. For both samples, the MBC/MIC ratios were > 4, which indicated probable bacteriostatic activities [28]. The best results observed for BoEA may be related to its higher phenolic and flavonoid content. BoEA showed a total phenolic content of 1554.75 ± 56.14 mg GAE/g and total flavonoid content of 146.55 ± 2.38 mg QE/g; these values were higher than those found for BoHE (1025.5 ± 66.33 mg GAE/g; 64.82 ± 2.05 mg QE/g).

### 3.2. Cytotoxic Evaluation of* B. orellana* Extracts

**Figure 1:**
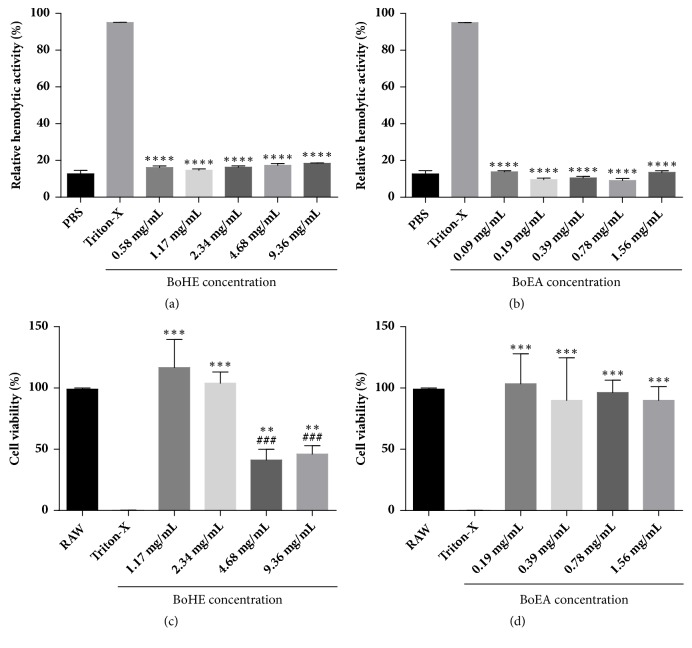
Cytotoxic effects of* Bixa orellana* leaves extracts (BoHE and BoEA). (a), (b) Hemolytic activity of* Bixa orellana* leaves extracts (BoHE and BoEA) performed with type A, B, and O human erythrocytes. (c), (d) Cytotoxicity of* Bixa orellana* leaves extract (BoHE and BoEA) towards RAW 264.7 cells determined by MTT assay. Results are means ± standard deviation of the results in triplicate. ^###^p < 0.0005 compared with untreated cells. *∗∗*p < 0.005; *∗∗∗*p < 0.0005 compared with Triton X. Each assay was performed in triplicate, and each experiment was repeated three times.

The cytotoxic effects of BoHE and BoEA towards erythrocyte and RAW 264.7 cells are shown in Figure 1. The extracts did not induce significant erythrocyte toxicity; the results were similar to those observed for erythrocytes treated with PBS (it was not possible to calculate IC50). Regarding the results with RAW 264.7 cells, the IC50 values were 7.44 mg/mL for BoHE and 7.716 mg/mL for BoEA. The selective index (SI, i.e., the ratio between IC50/MIC values) was 19.8 for BoEA and 3.2 for BoHE. The higher SI value for BoEA indicated a greater antimicrobial efficiency.

### 3.3. Effects of* B. orellana* Extracts on NO Production by RAW 264.7 Macrophages Infected with* M. abscessus massiliense*

**Figure 2:**
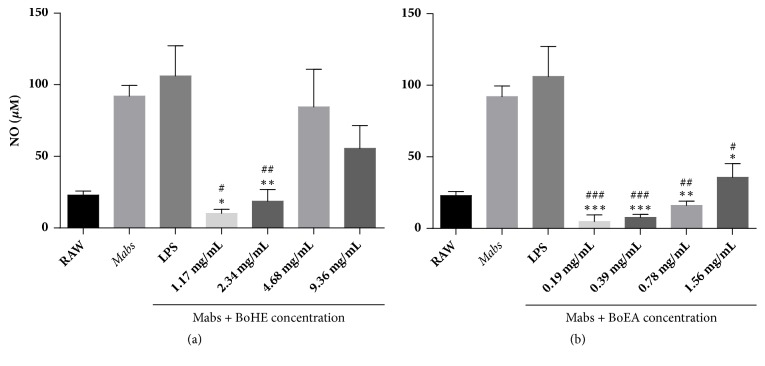
Effects of* B. orellana* leaves extract (BoHE and BoEA) on NO production by murine RAW 264.7 cells after infection with* M. abscessus *(*Mabs*). To evaluate NO production, RAW 264.7 cells were infected with 1 x 10^8^ CFU of* Mabs*, followed by treatment with BoHE or BoEA. Results are means ± standard deviation of the results in triplicate. *∗*p < 0.05; *∗∗*p < 0.005; *∗∗*p<0.0005 compared with the LPS control. The assay was performed in triplicate, and the experiment was repeated three times.

Figure 2 shows the effects of* B. orellana *extracts on NO production by RAW 264.7 macrophages infected by* Mabs*. As expected, the cells infected by* Mabs *exhibited high levels of NO in their supernatant, which were similar to the values found for the treatment with LPS. This effect was significantly inhibited by the treatment with all concentrations of BoEA (Figure 2(b)) and the lower concentrations of BoHE (0.19 mg/mL and 0.39 mg/mL) (Figure 2(a)).

### 3.4. *B. orellana* Extracts Inhibit Cell Migration Induced by* M. abscessus massiliense*

**Figure 3:**
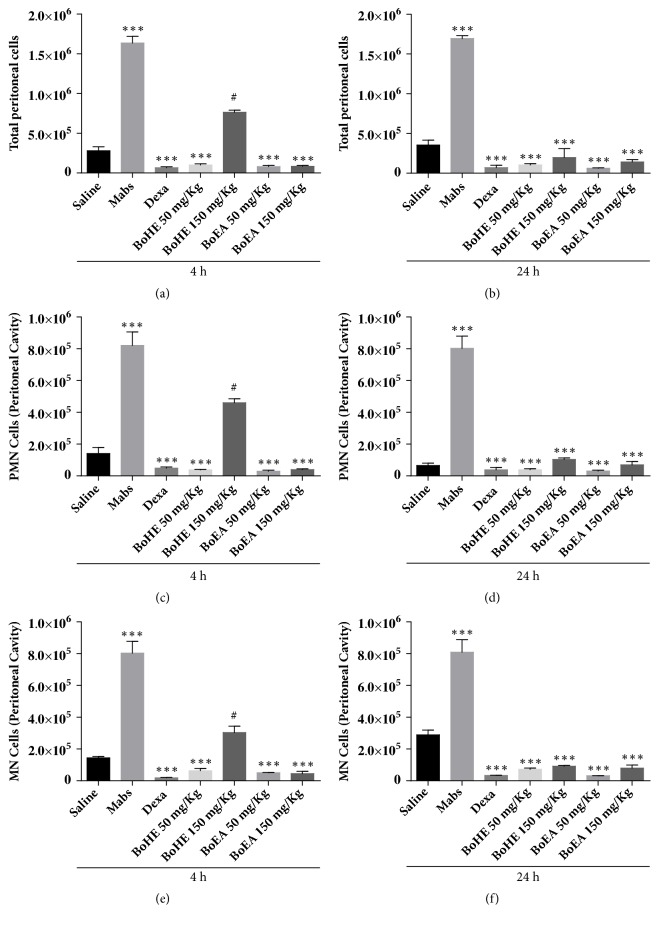
Effects of* Bixa orellana* extracts (BoHE and BoEA) on cell migration to peritoneal fluid after induction of peritonitis by heat killed* M. abscessus *(*Mabs*). Saline: animals treated with 0.9% saline solution (1 mL/kg) without* Mabs *inoculation (negative control);* Mabs*: animals treated with 0.9% saline solution (1 mL/kg) and inoculated with* Mabs*; Dexa: animals treated with 5 mg/kg dexamethasone and inoculated with* Mabs*; BoHE 50 mg/kg: animals treated with 50 mg/kg BoHE and inoculated with* Mabs*; BoHE 150 mg/kg: animals treated with 150 mg/kg BoHE and inoculated with* Mabs*; BoEA 50 mg/kg: animals treated with 50 mg/kg BoEA and inoculated with* Mabs*; BoEA 150 mg/kg: animals treated with 150 mg/kg BoEA and inoculated with* Mabs*. (a) Total leukocytes 4h after induction of peritonitis. (b) Total leukocytes 24h after induction of peritonitis. (c) Polymorphonuclear cells (PMN) 4h after induction of peritonitis. (d) Polymorphonuclear cells (PMN) 24h after induction of peritonitis. (e) Mononuclear cells (MN) 4h after induction of peritonitis. (f) Mononuclear cells (MN) 24h after induction of peritonitis. *∗∗∗*p < 0.001 compared with* Mabs* controls. ^#^p < 0.001 compared with saline controls.

The anti-inflammatory effects of BoHE and BoEA were evaluated in an experimental mouse peritonitis model induced by intraperitoneal injection of heat killed* Mabs*. As shown in Figure 3, high numbers of leukocytes were detected in the peritoneal fluid of animals inoculated with heat killed* Mabs *after 4 h and 24 h. This effect was inhibited in animals treated with BoHE or BoEA. After 4 h of peritonitis, the best results were observed for animals treated with both doses of BoEA (0.81 ± 0.38 x 10^5^ cells/mL for 50 mg/kg and 0.89 ± 0.27 x 10^5^ cells/mL for 150 mg/kg); these results were similar to those observed in animals treated with dexamethasone (0.6 ± 0.33 x 10^5^ cells/mL). The maximum BoHE inhibition was found at 50 mg/kg (1.01 ± 0.3 x 10^5^ cells/mL), while the dose of 150 mg/kg induced a weak effect (7.64 ± 0.58 x 10^5^ cells/mL) (Figure 3(a)).

Similar results were observed after 24 hours of inflammation induction (Figure 3(b)). When BoEA was inoculated at 50 mg/Kg, the greatest reduction on total leukocytes levels were observed (0.62 ± 0.15 x 10^5^ cells/mL), followed by the pretreatment with BoHE at 50 mg/kg (1.04 ± 0.3 x 10^5^ cells/mL), BoEA at 150 mg/kg (1.4 ± 0.65 x 10^5^ cells/mL), and BoHE at 150 mg/kg group (1.98 ± 0.2 x10^5^ cells/mL). The inhibition in response to BoEA at 50 mg/kg was stronger than that in response to dexamethasone (0.71 ± 0.07 x 10^5^ cells/mL).

Regarding the number of polymorphonuclear cells (PMN) in the peritoneal lavage fluid (Figure 3(c)), after 4 hours the best results were also found for animal pretreated with BoEA (0.29 ± 0.14 x 10^5^ cells/mL for BoEA at 50 mg/kg, and 0.39 ± 0.13 x 10^5^ cells/mL for 150 mg/kg); however, BoHE administration also induced strong results (50 mg/kg= 0.38 ± 0.063 x 10^5^ cells/mL; 150 mg/kg = 4.6 ± 0.49 x 10^5^ cells/mL). All these values were significantly different when compared with the PMN amount found for untreated animal inoculated with heat killed* Mabs *(8.2 ± 1.7 x 10^5^ cells/mL). In addition, the results were similar to those in dexamethasone-treated controls (0.48 ± 0.2 x 10^5^ cells/mL), except for BoHE at 150 mg/kg. The number of PMN remained high 24 h after* Mabs *inoculation (8.02 ± 1.7 x 10^5^ cells/mL) and was reduced in animals treated with the extracts, particularly those treated with 50 mg/kg (0.3 ± 0.12 x 10^5^ cells/mL) and 150 mg/Kg (0.69 ± 0.51 x 10^5^ cells/mL) BoEA. These results were similar to those observed in dexamethasone-treated animals (0.3 ± 0.04 x 10^5^ cells/mL) (Figure 3(d)).

Finally, the effects of BoEA and BoHE on mononuclear cells (MNs) migration induced by* Mabs *inoculation were also determined (Figures 3(e) and 3(f)). At 4 h after the induction of peritonitis, the number of PMN was higher in the* Mabs* (8.04 ± 1.47 x 10^5^ cells/mL) than in the saline group (1. 44 ± 0.19x10^5^ cells/mL) (Figure 3(e)). MN migration was inhibited most strongly in animals pretreated with 50 mg/kg BoEA (0.51 ± 0.03 x 10^5^ cells/mL), followed by the doses of 150 mg/kg BoEA (0.96 ± 0.45 x 10^5^ cells/mL), 50 mg/kg BoHE (1.17 ± 1.03 x 10^5^ cells/mL), and 150 mg/kg BoHE (3.03 ± 0.9 x 10^5^ cells/mL). As shown in Figure 3(f), this effect continued through 24 h, when inhibition was strongest in the pretreatment with 50 mg/kg BoEA (0.32 ± 0.02 x 10^5^ cells/mL), followed by 50 mg/kg BoHE (0.75 ± 0.21x10^5^ cells/mL), 150 mg/kg BoEA (0.81 ± 0.54 x 10^5^ cells/mL), and BoHE (0.92 ± 0.1 x 10^5^ cells/mL).

### 3.5. Acute Toxicity

**Figure 4:**
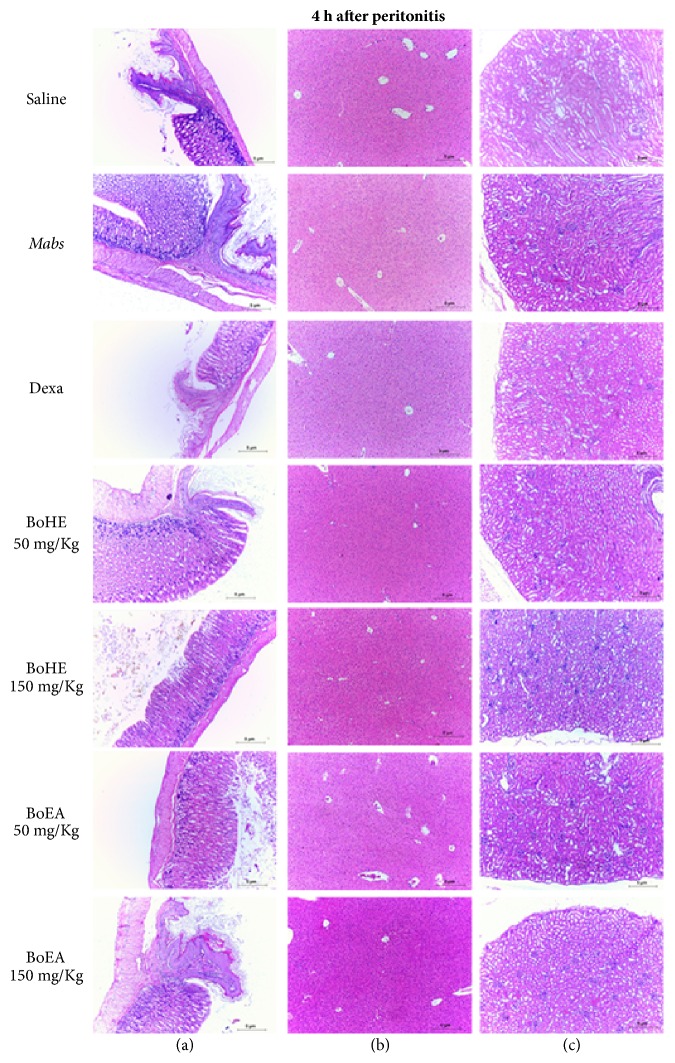
Evaluation of the toxicity of* Bixa orellana* extracts (BoHE and BoEA) in C57BL/6 mice 4 h after induction of peritonitis. Saline: animals treated with 0.9% saline solution (1 mL/kg) without* Mabs *inoculation (negative control);* Mabs*: animals treated with 0.9% saline solution (1 mL/kg) and inoculated with* Mabs*; Dexa: animals treated with 5 mg/kg dexamethasone and inoculated with* Mabs*; BoHE 50 mg/kg: animals treated with 50 mg/kg BoHE and inoculated with* Mabs*; BoHE 150 mg/kg: animals treated with 150 mg/kg BoHE and inoculated with* Mabs*; BoEA 50 mg/kg: animals treated with 50 mg/kg BoEA and inoculated with* Mabs*; BoEA 150 mg/kg: animals treated with 150 mg/kg BoEA and inoculated with* Mabs*. Column (A): esophageal/stomach transition area with no significant histological changes. Column (B): liver histology has a normal appearance. Column (C): kidney histology has a normal appearance. H&E stain, ×100.

**Figure 5:**
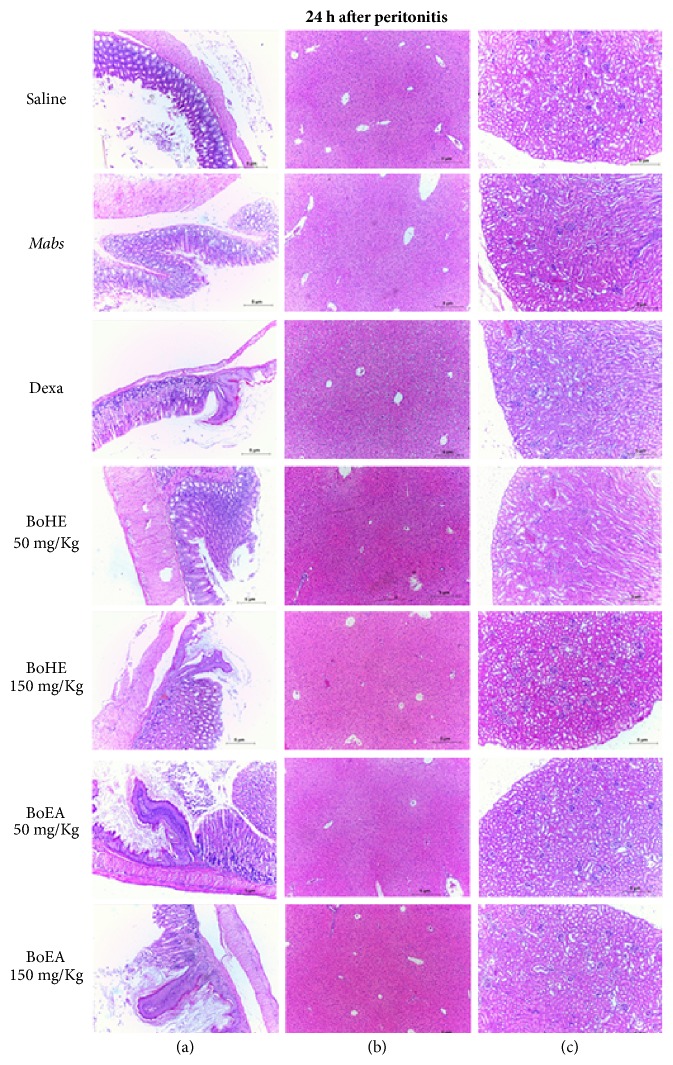
Evaluation of the toxicity of* Bixa orellana* extracts (BoHE and BoEA) in C57BL/6 mice 24 h after induction of peritonitis. Saline: animals treated with 0.9% saline solution (1 mL/kg) without* Mabs *inoculation (negative control);* Mabs*: animals treated with 0.9% saline solution (1 mL/kg) and inoculated with* Mabs*; Dexa: animals treated with 5 mg/kg dexamethasone and inoculated with* Mabs*; BoHE 50 mg/kg: animals treated with 50 mg/kg BoHE and inoculated with* Mabs*; BoHE 150 mg/kg: animals treated with 150 mg/kg BoHE and inoculated with* Mabs*; BoEA 50 mg/kg: animals treated with 50 mg/kg BoEA and inoculated with* Mabs*; BoEA 150 mg/kg: animals treated with 150 mg/kg BoEA and inoculated with* Mabs*. Column (A): esophageal/stomach transition area with no significant histological changes. Column (B): liver histology has a normal appearance. Column (C): kidney histology has a normal appearance. H&E stain, ×100.

The evaluation of acute toxicity included the histological evaluation of esophagus, stomach, liver, and kidney tissue samples from all experimental groups used in this study. No significant histological changes were observed at either 4 h or 24 h. Normal tissue and organ structures were maintained (Figures 4 and 5). The maintenance of esophageal and gastric mucosa confirmed the satisfactory performance of the therapeutic gavage protocol. The architecture of the evaluated organ tissues was preserved, with normal histological characteristics.

### 3.6. HPLC-MS Analysis of BoEA

**Figure 6:**
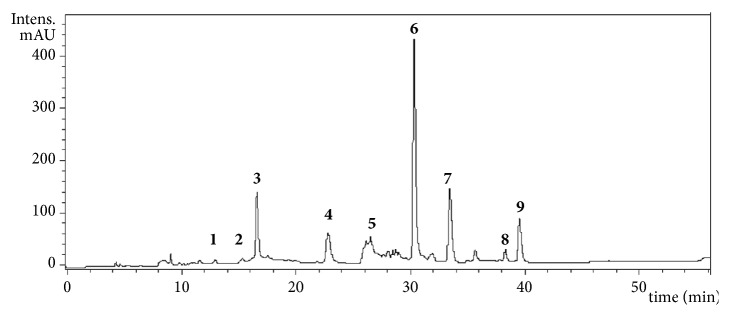
HPLC/DAD chromatogram of the acetyl acetate extract of* Bixa orellana* leaves (BoEA) monitored at 254 nm. Structure of constituents identified by HPLC3 DAD-ESI-IT/MS. ID: identification; RT: retention time; MS: mass spectrometer.

Figure 6 illustrates the HPLC-DAD chromatogram of the phenolic compounds from ethyl acetate phase from hydroalcoholic extract from leaves of* B. orellana.* A total of nine peaks corresponding to compounds 1–9 were tentatively identified on the basis of their retention times and MS pattern with also taking into account data in the related literature. These compounds included procyanidin B-2 and B-3, granatin B, neostrictinin, ellagitannin isomer, kaempferol-3-O-*β*-D-6-(p-coumaroyl) glucopyranoside, ellagic acid glucoside, kaempferol-3-O-D-glucoside, and ellagic acid deoxyhexose as shown in Table 1.

## 4. Discussion

This study evaluated the antimicrobial and anti-inflammatory activities of* B. orellana*, a species widely used in folk medicine for the treatment of infections, headache, dysentery, fever, indigestion, and skin diseases [29, 30]. The tested* B. orellana* extracts had antimicrobial activity against* M. abscessus*. Although* B. orellana* is widely used to treat respiratory infections, this is the first report of its kind. The study results are consistent with a previous study reporting MIC values of 0.3, 0.5, and 0.2 mg/mL for* B. orellana *leaf, seed, and root extracts against* M. tuberculosis *[31]. Only few studies have described the antimycobacterial activity of plant-derived products against* M. abscessus, *for example, the effects of chloroform and ethanolic extracts from seeds of* Persea americana* (where the activity of the extracts was associated with lignans) [32], Valencia orange terpeneless oil [33], and* Pelargonium reniforme and P. sidoides *root extracts (containing linear chain fatty acids such as palmitic, oleic, and linoleic) [34].

Inflammatory responses play important roles in host defense and also contribute to immunopathogenesis during mycobacterial infection [35].* M. abscessus* is a respiratory pathogen causing chronic lung diseases and infections associated with cystic fibrosis [36]. A strain of* M. abscessus* forms colonies with rough morphology and is known to induce inflammatory responses associated with invasive disease [37]. The anti-inflammatory activity of BoHE and BoEA was evaluated in a C57BL/6 mouse model of peritonitis induced by heat killed* M. abscessus*.* M. abscessus* is an etiological agent of infections associated with laparoscopic gastric bands [38] and causes acute peritonitis that can evolve with ascites and formation of granulomas [39].

In the study model, oral pretreatment with BoHE or BoEA (both at 50 mg/kg or 150 mg/kg) significantly reduced the migration of leukocytes to the peritoneal cavity. The observation that the effects were similar to those observed with the anti-inflammatory dexamethasone suggests that the extracts had secondary metabolites with anti-inflammatory activity. The anti-inflammatory activity of* B. orellana-*derived products has been previously reported [19, 20, 40].

In this study, BoHE and BoEA reduced NO production in* Mabs*-infected RAW 264.7 murine macrophages. It has been shown that peritoneal macrophages of C57BL/6 mice produce high levels of NO after infection with* M. abscessus* [41]. NO produced by activated cells has cytotoxic and microbicidal action that promotes the destruction of invading microorganisms [42]. BoHE and BoEA might reduce NO production by inhibiting enzymes responsible for the production of induced nitric oxide synthase (iNOS) [43].

The migration of leukocytes into inflamed tissue from the microcirculation is characteristic of acute inflammation [44]. The anti-inflammatory activity of BoHE and BoEA extracts was evaluated in a model of peritonitis induced by heat killed* M. abscessus* in which treatment with the extracts reduced the peritoneal migration of leukocytes. The BoHE and BoEA extracts were both protective against the development of acute inflammation in the mouse model used in this study. The* B. orellana *active extract contained several flavonoids, such as kaempferol, ellagic acid, and HHDP glucose, which are known immunomodulators [45–49].

Determining the toxicity of natural products with therapeutic potential is fundamental to the investigation of their bioactive potential. Effective and safe therapeutics are more active against pathological agents than the cells and tissues of the organism being treated. Histological analysis of esophagus, stomach, liver, and kidney tissue confirmed that the BoHE and BoEA treatment regimens, the concentration, the route of administration, and the exposure time were neither hepatotoxic nor nephrotoxic. These are essential criteria when evaluating new therapeutic compounds. Oral administration, as used in this study, did not disturb the normal histology of the esophageal or gastric mucosa. The absence of mucosal damage allows for drug absorption and satisfactory biotransformation, both of which are essential [50].

## 5. Conclusions

The extracts of* B. orellana* leaves (BoHE and BoEA) had antimicrobial activity against* M. abscessus *subsp.* massiliense *and low toxicity against murine RAW 264.7 cells. The antimycobacterial activity was accompanied by significant anti-inflammatory activity in a peritonitis model induced by* M. abscessus *subsp.* massiliense* in mice. Inhibition of the migration of leukocytes to the site of inflammation was associated with secondary metabolites. The extracts did not cause acute toxicity in the evaluated organs. Taken together, the results of the study demonstrated the antimicrobial and anti-inflammatory potential of the BoHE and BoEA extracts of* B. orellana*, which makes these natural compounds targets for drug development. Further* in vivo *studies of the antimicrobial effect of BoHE and BoEA are warranted.

## Figures and Tables

**Table 1:** Spectrometric data of phenolic compounds identified in the ethyl acetate extract of *Bixa orellana* leaves (BoEA).

**Peak**	**R**_**t**_	**Compound**	**[M-H]** ^−^	**MS** ^**n**^ ** fragments**
1	12.8	procyanidin B-2	577	559, 451, 425, 407, 289
2	14.5	procyanidin B-3	577	559, 451, 425, 407, 289
3	16.5	granatin B	951	933
4	23.1	neostrictinin	633	463, 301, 275
5	26.3	ellagitannin isomer	953	935, 301
6	31.0	kaempferol-3-O-*β*-D-6-(*p*- coumaroyl) glucopyranoside	593	285
7	33.1	ellagic acid glucoside	463	301
8	38.1	kaempferol-3-O-D-glucoside	447	285
9	39.0	ellagic acid deoxyhexose	477	301

R_t_ expressed in min; [M - H]^−^ and MS^n^ fragments in *m/z*.

## Data Availability

The values used to build graphs to support the findings of this study are available from the corresponding author upon request.
